# Seroprevalence and associated factors for hepatitis B and hepatitis C viral infection among patients with diabetes mellitus in Northern Tanzania

**DOI:** 10.1371/journal.pone.0319198

**Published:** 2025-02-20

**Authors:** Doreen T. Eliah, Nyasatu G. Chamba, Abid M. Sadiq, Amos O. Mwasamwaja, Faustini C. Kimondo, Cuthbert D. Matay, Eliada B. Nziku, Tumaini E. Mirai, Ibrahim Ali Ibrahim Muhina, Fuad H. Said, Sarah K. Gharib, Furaha S. Lyamuya, Elifuraha W. Mkwizu, Kajiru G. Kilonzo, Venance P. Maro, Elichilia R. Shao

**Affiliations:** 1 Department of Internal Medicine, Kilimanjaro Christian Medical University College, Moshi, Tanzania; 2 Department of Internal Medicine, Kilimanjaro Christian Medical Center, Moshi, Tanzania; 3 Division of Gastroenterology, Department of Medicine, Aga Khan University Hospital, Nairobi, Kenya; Centers for Disease Control and Prevention, UNITED STATES OF AMERICA

## Abstract

**Background:**

The coexistence of viral hepatitis with diabetes mellitus (DM) significantly escalates the risk of severe outcomes. This study aimed to determine the seroprevalence and associated factors of hepatitis B (HBV) and hepatitis C (HCV) viral infections among DM patients in northern Tanzania.

**Materials and methods:**

Conducted between February 2023 and May 2023, this hospital-based cross-sectional study enrolled 189 patients with DM from the Diabetic Clinic of Kilimanjaro Christian Medical Centre. A structured questionnaire captured relevant clinical information, and plasma blood sample was assessed for hepatitis B surface antigen and anti-hepatitis C antibody seropositivity. Data analysis employed SPSS v26, and a chi-square test was used to determine the statistical difference of HBV and HCV among patients with DM. Logistic regression was performed to determine factors associated with HBV and HCV.

**Results:**

Among the 189 patients with DM, the seroprevalence of HBV and HCV infections stood at 2.1% and 0.5%, respectively. Males constituted a significant majority (80%) of those affected by viral hepatitis. Furthermore, 60% of affected patients were in non-union relationships (single, widowed, or divorced), and 40% reported multiple sexual partners. However, the study found no significant association between traditional associated factors and viral infection acquisition.

**Conclusion:**

The study’s findings reveal a relatively low prevalence of HBV and HCV infections among patients with DM compared to the general population, with no significant association among factors. Nonetheless, the results underscore the importance of early screening and vaccination for HBV and HCV in patients with DM. Such efforts are crucial for curbing infection spread and reducing the risk of hepatocellular carcinoma development in this vulnerable population.

## Introduction

Hepatitis B (HBV) and Hepatitis C (HCV) viral infections are globally estimated to infect 350 million and 170 million people, respectively, which accounts for about 96% of the 1.3 million deaths of all viral hepatitis infections [1,2]. Among cirrhotic patients, the estimated prevalence of HBV and HCV was 42% and 21%, respectively, with more viral hepatitis infections widely distributed among the Asian and African continents [3].

Diabetes mellitus (DM) affects about 463 million people globally, accounting for over 1.5 million deaths [4]. In Africa, it was estimated that about 24 million people were living with DM in 2021, and Tanzania is among the leading countries in sub-Saharan Africa, having a prevalence of 12.3% in 2021 [5].

DM, a chronic metabolic disorder, is an independent risk factor in the development of end-stage liver disease and hepatocellular carcinoma through a metabolic syndrome associated with non-alcoholic fatty liver disease [6]. Patients with DM are in an immunocompromised state, increasing the risk of chronically being infected by viral hepatitis. Nevertheless, this group is also at risk of acquiring the infection from parenteral exposures due to recurrent admission, hemodialysis, blood transfusion, surgical and dental intervention, and the day-to-day sharing of sharp objects during glucose monitoring.

HBV and HCV can present with intrahepatic and extrahepatic manifestations resulting from the host’s interplay between innate and adaptive immunity. This leads to liver necrosis, end-stage liver disease, and other tissue damage causing cardiovascular, nephrotic, neuropathic, and metabolic manifestations. The development of metabolic manifestations, including hyperglycemia and insulin resistance, impairs glycemic control and exacerbates both macro- and micro-complications of DM [7].

It is noteworthy to mention that there may be a two-way relationship between DM and HCV infection. Patients with DM may be 3.5 times more likely to acquire an HCV infection. Conversely, HCV infection is associated with an approximately 1.5-fold excess risk of new-onset T2DM development. Theories speculate that insulin resistance positively correlates with HCV viral load. Additionally, insulin resistance and DM may be associated with metabolic extrahepatic manifestations of chronic HCV infection [8]. Similar with HBV leading to increased risk of hepatocellular carcinoma in the presence of DM [9]. By screening for viral hepatitis among patients with DM, morbidities associated with HBV and HCV infections and DM are significantly attenuated, resulting in reduced healthcare costs attributed to the diseases and their corresponding complications [10].

In Tanzania, the estimated prevalence of HBV is around 6.9%, highest in the northern zone (9.3%), and lowest in the lake zone (4.6%), suggesting a moderately high endemicity [11]. However, the mortality rate is estimated to be 6.95 per 100,000 people [12]. The estimated prevalence of HCV is around 2.9%, with an estimated mortality rate of 5.49 per 100,000 people [12].

Given the risk and complications of HBV and HCV in patients with DM, screening among this group is crucial, as little is known about the burden of HBV and HCV among patients with DM, especially in Tanzania, which is necessary for intervention strategies. At Kilimanjaro Christian Medical Centre (KCMC), out of 3.930 patients during a one-year inpatient assessment, 1.3% with HBV and 0.2% with HCV were admitted with complications, as six and three patients, respectively, had DM [13]. The aim of this study was to determine the seroprevalence of HBV and HCV and the associated factors associated with patients with DM.

## Materials and methods

### Study design and setting

The study was a hospital-based cross-sectional study involving patients with DM attending the diabetic clinic at KCMC from February 2023 to May 2023. The diabetic clinic is one of the outpatient clinics under the department of internal medicine, which is conducted once a week and accommodates up to 60 adult patients per day. Sampling and recruitment were done voluntarily, and 189 patients with DM were enrolled in the study. DM was defined in patients with a history of DM diagnosis, currently on treatment, or a fasting blood glucose ≥7.0 mmol/L. The inclusion criteria were all patients with DM, both type 1 and type 2, aged 18 years and older, who were attending the diabetic clinic at KCMC and had consented to participate. However, pregnant women with gestational diabetes and patients with DM who were frail and unable to give the necessary information were excluded.

A minimum sample size for this study was estimated based on a study done in Nigeria that looked at viral hepatitis among patients with DM [14]. Therefore, a hypothetical proportion of 13.3% was used to estimate the sample size by using the Cochran equation, N = (Z^2^pq)/(E^2^). The minimum sample size required at KCMC was 177 patients. Taking into consideration the 10% non-response rate, the total sample size was 195. As the diabetic clinic was operated once a week, all patients were informed of the study and those interested volunteered to be part of the study, implementing a consecutive sampling technique to recruit patients.

### Ethical considerations

Permission to conduct the study was sought from the Kilimanjaro Christian Medical College Research Ethics and Review Committee with clearance number PG 55/2022 and from the head of the department of internal medicine. Written consent was obtained from the patients after they were informed of the purpose of the study. Participation was voluntary, and there was no impact on the treatment of the patient as they were not denied their right to medical care. Confidentiality was observed, and all data was stored unlinked to patient identifiers as each patient had an ID number and not names to abide by confidentiality.

### Data and sample collection

Data was collected using a structured questionnaire and filled out during a face-to-face interview. The questionnaire contained two parts: the first part included socio-demographic characteristics, and the second part included clinical characteristics and the associated factors of the patients.

From all the patients, a finger stick prick was done to draw two drops of blood using a pipette to measure blood glucose using the GlucoNavii glucometer, and a blood glucose of ≥10 mmol/L was considered uncontrolled according to American Diabetes Association criteria. For the viral serology, two drops of blood from a finger prick using disposable prickers were collected using a disposable pipette to test for hepatitis B surface antigen using the DIAGNOSTAR rapid test and for anti-hepatitis C antibody using the LABOREX diagnostic rapid test. All are chromatographic immunoassay kits, with procedures followed according to the manufacturer’s protocol.

All the patients that tested positive had their phone numbers taken and were assigned and followed up in the diabetes clinic for confirmation tests and treatment. And the negative test patients were given health information about HBV and HCV and also advised on vaccination against HBV. Data from the patient was uploaded and stored electronically using the SurveyCTO software application.

### Statistical analysis

The data was downloaded to SPSS version 26 for cleaning and analysis. Quantitative variables were summarized into mean, median, and measure of dispersion, while frequency and percentage for categorical variables were presented into frequency tables. In a bivariate analysis, the Fisher’s exact test was used (most expected cell counts are < 5) to determine the statistical difference of HBV and HCV among patients with DM, and categorical variables through contingency tables were considered to be associated. The logistic regression was performed to determine factors associated with HBV and HCV. Covariates with a p-value < 0.1 in the bivariate analysis were included in the multivariate logistic regression model. The association was considered statistically significant at a 95% confidence interval and a p-value of < 0.05.

## Results

A total of 195 patients were identified, but six patients refused to have their blood sample taken during the study period. A total of 189 patients were enrolled in this study, with a mean age of 62 years (SD 11.2). Among the patients, 118 (62.4%) were female, 118 (62.4%) were in a union, 121 (64%) resided in rural areas, and 96 (50.8%) had primary level education. With regards to employment, 61 (32.3%) were unemployed, and among the patients employed, 21 (11%) were healthcare workers (Table 1).

**Table 1:** Participants socio-demographic characteristics of study participants (N = 189).

Variables	n	%
Age (years); *mean (SD) 62.0 (11.20)*		
≤50	25	13.2
>50	164	86.8
Sex		
Male	71	37.6
Female	118	62.4
Marital status		
Union (Married/Cohabiting)	118	62.4
Nonunion (Single, Divorced, Widowed)	71	37.6
Education level		
Primary level	96	50.8
Secondary level	36	19.0
Tertiary level	57	30.2
Residence		
Rural	121	64.0
Urban	68	36.0
Employment		
Formal employment	33	17.5
Self-employed	49	25.9
Unemployed	61	32.3
Retired	46	24.3
Working in healthcare sector		
No	168	88.9
Yes	21	11.1
Number of sexual partners		
One	152	80.4
More than one	37	19.6
Alcohol consumption		
No	80	42.3
Yes	109	57.7

Among the clinical characteristics of the patients, 174 (92.1%) had type 2 DM, 106 (56.1%) were using oral medication, and the majority of the patients (63.7%) had normal blood sugar levels with a median of 9.6 mmol/L (IQR 6.9–13.0). With regards to vaccination status, the majority of the patients (74.1%) had not been vaccinated for HBV. Eighty-seven (46%) had recently been admitted to the hospital, and 57 (30.2%) had a history of recent minor or major surgery. Fifty-four (28.6%) had undergone a dental procedure, 32 (6.9%) were on hemodialysis, 51 (27%) have a history of blood transfusion, and 21 (11.1%) have had a tattoo or piercing on their body (Table 2).

**Table 2:** Clinical characteristics of the study participants (N = 189).

Variables	n	%
Type of DM		
Type 1	15	7.9
Type 2	174	92.1
Type of medication		
Oral	106	56.1
Injections	21	11.1
Both	62	32.8
Recent admission		
No	102	54.0
Yes	87	46.0
Recent surgical intervention		
No	132	69.8
Yes	57	30.2
History of dental procedure		
No	135	71.4
Yes	54	28.6
Hemodialysis initiation		
No	157	83.1
Yes	32	16.9
Piercing and tattoo		
No	168	88.9
Yes	21	11.1
Sharing of sharp objects		
No	186	98.4
Yes	3	1.6
Blood transfusion history		
No	138	73.0
Yes	51	27.0
Hepatitis B vaccine		
No	140	74.1
Yes	49	25.9
RBG (mmol/L); *median (IQR) 9.60 (6.90–13.00)*		
≥10	69	36.5
4–10	120	63.5
Hepatitis serology		
Positive	5	2.6
Negative	184	97.4
Hepatitis strain		
Hepatitis B	4	2.1
Hepatitis C	1	0.5

Among the patients, seroprevalence of HBV was 2.1% (4/189; 95% CI 2.08–2.12) and HCV was 0.5% (1/189; 95% CI 0.49–0.51); there was no case for coinfection of HBV and HCV. The majority of the seropositive patients (those having either HBV or HCV positivity) were males, accounting for about 80%. About 2/5 (40%) of the patients had more than one sexual partner. The proportion of seropositive patients varies across socio-demographic and clinical characteristics among patients (Table 3).

**Table 3:** Hepatitis B and C serology by participant’s characteristics (N = 189).

Variable	Hepatitis B & C		cOR (95% CI)	p value	aOR (95% CI)	p value
	Positive	Negative				
Age (years)						
≤50	2 (40.0)	23 (12.5)	4.67 (0.74–29.44)	0.131		
>50	3 (60.0)	161 (87.5)	1			
Sex						
Male	4 (80.0)	67 (36.4)	6.99 (0.77–63.79)	0.067	5.68 (0.58–55.33)	0.135
Female	1 (20.0)	117 (63.6)	1			
Marital status						
Non-Union (Single/Divorced/Widowed)	2 (40.0)	69 (37.5)	1.11 (0.18–6.82)	0.622		
Union (Married/Cohabiting)	3 (60.0)	115 (62.5)	1			
Education level						
Primary level	2 (40.0)	94 (51.1)	0.59 (0.08–4.27)	0.597		
Secondary level	1 (20.0)	35 (19.0)	0.79 (0.07–8.99)	0.846		
Tertiary level	2 (40.0)	55 (29.9)	1			
Residence						
Rural	3 (60.0)	118 (64.1)	0.84 (0.14–5.15)	0.592		
Urban	2 (40.0)	66 (35.9)	1			
Employment						
Formal employment	2 (40.0)	31 (16.9)	1.52 (0.20–11.34)	0.685		
Self-employed	2 (40.0)	47 (25.5)	3.87 (0.34–44.38)	0.277		
Unemployed	1 (20.0)	60 (32.6)	–	1.000		
Retired	0 (0.0)	46 (25.0)	1			
Working in health care sector						
No	5 (100)	163 (88.6)	0.97 (0.95–1.00)	1.000		
Yes	0 (0)	21 (11.4)	1			
Number of partners						
One	3 (60.0)	149 (81.0)	1			
More than one	2 (40.0)	35 (19.0)	2.84 (0.46–17.63)	0.253		
Alcohol consumption						
No	1 (20.0)	79 (42.9)	1			
Yes	4 (80.0)	105 (57.1)	3.01 (0.33–27.45)	0.398		
Type of DM						
Type 1	2 (40.0)	13 (7.1)	8.76 (1.35–57.24)	0.051	4.46 (0.24–81.38)	0.313
Type 2	3 (60.0)	171 (92.9)	1			
Type of medication						
Oral	2 (40.0)	104 (56.5)	1			
Injections	2 (40.0)	19 (10.3)	5.01 (0.92–31.56)	0.063	2.49 (0.43–49.34)	0.203
Both	1 (20.0)	61 (31.2)	0.50 (0.05–4.61)	0.544	0.54 (0.03–11.30)	0.688
Recent admission						
No	2 (40.0)	100 (54.3)	1			
Yes	3 (60.0)	84 (45.7)	1.79 (0.29–10.94)	0.663		
Recent surgical intervention						
No	3 (60.0)	129 (70.1)	1			
Yes	2 (40.0)	55 (29.9)	1.56 (0.25–9.62)	0.639		
History of dental procedure						
No	4 (80.0)	131 (71.2)	1			
Yes	1 (20.0)	53 (28.8)	0.62 (0.07–5.66)	0.557		
Hemodialysis initiation						
No	4 (80.0)	153 (83.1)	1			
Yes	1 (20.0)	31 (16.9)	1.23 (0.13–11.42)	0.609		
Piercing and/or tattoo						
No	3 (60.0)	165 (89.7)	1			
Yes	2 (40.0)	19 (10.3)	5.79 (0.91–36.86)	0.096	4.82 (0.60–39.04)	0.141
Sharing of sharp objects						
No	5 (100)	181 (98.4)	1			
Yes	0 (0)	3 (1.6)	0.97 (0.95–1.00)	1.000		
Blood transfusion history						
No	4 (80.0)	134 (72.8)	1			
Yes	1 (20.0)	50 (27.2)	0.67 (0.07–6.14)	0.591		

DM: Diabetes mellitus.

Regarding the factors associated with seropositivity to HBV or HCV, the factors mentioned included multiple sexual partners, recent surgical and dental interventions, recent admissions, hemodialysis, the sharing of sharp objects, having tattoos, and drinking alcohol. Males had an approximate 7-fold odds of viral hepatitis in DM (cOR 6.99; 95% CI: 0.77–63.79; p = 0.067). Patients with type 1 DM had an 8.7-fold odds of viral hepatitis (cOR 8.76; 95% CI: 1.35–57.24; p = 0.051). Patients only on insulin had a 5-fold odds of viral hepatitis (cOR 5.01; 95% CI: 0.92–31.56; p = 0.063). Patients who had a piercing and/or tattoo had an approximate 6-fold odds of viral hepatitis (cOR 5.79; 95% CI: 0.91–36.86; p = 0.096). On multivariate analysis, none of the variables were found to be statistically significant (Table 3).

## Discussion

This cross-sectional study recruited 189 patients who were attending the diabetic clinic at KCMC. The seroprevalence among the patients with DM for HBV and HCV was 2.1% (95% CI 2.08–2.12) and 0.5% (95% CI 0.49–0.51), respectively. However, among the associated factors analyzed, there was no significant association with the outcome of interest, which may be due to the small number of seropositive patients.

The seroprevalence of HBV and HCV in this study was lower than that of the general population in Tanzania, which was pooled to be 6.91% (95% CI 5.18–8.86) for HBV [11] and 3.2% (95% CI 0.5–8.6) for HCV [15]. These results were not expected considering the increased risk of acquiring infection among patients with DM. Moreover, the seroprevalence of HBV among different social groups was higher than this study’s seroprevalence. These groups included pregnant women (8.0% (95% CI: 5.0–12.1) in Dar-es-Salaam), children (4.2% (95% CI 2.5–5.9) in Kilimanjaro), and blood donors nationwide, where the seroprevalence was 6.2% (12,198/196,735) [16], and among healthcare workers, the risk was higher with a seroprevalence of 5.7% (25/442) in Kilimanjaro [17]. One can argue that the high seroprevalence of HBV in these populations can be explained by less screening and poor uptake of vaccination against HBV, which can be attributed to the knowledge gap on why they should vaccinate despite HBV, vaccine availability, and inadequate safety control measures among these groups, exposing them to a higher risk of acquiring HBV [16,17]. Also, intra-regional differences in prevalence could account for the different seroprevalences.

Nevertheless, in regards to HBV, similar findings were noted in studies done in Iraq [18] and the USA [19], where the prevalence was 2.1% (8/375) and 2.3% (40/1,709), respectively. However, the study in Iraq excluded type 1 DM patients and was also a monocenter study involving a private clinical setting [18], which could suggest the low seroprevalence as compared to the higher seroprevalence estimated in the Asian population. The study in the USA, despite employing the same methodology, recruited a larger number of patients [19], making it more representative. This can put the emphasis on screening for HBV and vaccine uptake, increasing the seroprevalence.

Among African countries, the seroprevalence of HBV was higher in the DRC (3.4%; 5/149) [20], Ethiopia (8.5%; 26/610) [21], and Nigeria (13.3%; 24/180) [14]. In Asian countries, the seroprevalence was higher in South Korea (3.8%; 16,707/439,708) [22], Taiwan (13.5%; 111/820) [23], and Turkey (3.7%; 47/1,263) [24]. The difference may be attributed to the methodological aspects, including the screening tools more commonly employed (ELISA) and the fact that more female study patients were HBV positive.

For HCV, the same seroprevalence as the study was demonstrated by the study done in Iraq, which was 0.5% (2/375) [18]. However, in most African and Asian countries, the seroprevalence of HCV was higher in the DRC (24.6%; 37/149 [20] and 10.6%; 19/180 [25]), Egypt (31.3%; 71/227) [26], Cameroon (17.6%; 95% CI 14.0–21.2) [27], Ethiopia (7.5%; 23/305) [21], and Taiwan (6.8%; 56/820) [23]. In comparison to the study, all the above studies had a larger number of patients with different characteristics, including mean age distribution and sex distribution, where females were more affected.

In this study, the associated factors tested showed no association with the acquisition of the viral infection among the patients. This can be explained by the small number of patients who were seropositive in the regression analysis of the findings. Nevertheless, from the literature, there are conflicting results, as some studies suggest an association between parenteral exposures and viral infection among DM patients. For example, a study done in Ethiopia among patients with DM showed an association with an increased risk of acquiring HBV or HCV with recent admission, a recent surgical procedure, and the sharing of needles to acquire the viral infection [21]. Moreover, some studies showed a significant association with alcohol consumption, which was linked to risky behavior in alcohol-consuming patients with DM [14,18]. However, some showed no association, like the study done in Iraq, which showed no association with parenteral access [23]. This can demonstrate that geographical location can influence risk factors for seroprevalence of HBV and HCV, even among DM patients.

The limitations of this study were that it included a small sample, which may not have found any association, and a much larger study would be recommended. It only did the serological marker and used chromatography immunoassay as compared to most studies that used ELISA, which may suggest some variation in seroprevalence. Also, we could not identify whether the viral infection was acute or chronic at the point of testing, which needs further molecular confirmation. The study was done in a single center, which might suggest low seroprevalence among patients with DM in this region, which may not be generalized considering the interregional difference in seroprevalence. A larger study that incorporates baseline liver and glycated hemoglobin parameters would be beneficial in evaluating the relationship and control between viral hepatitis and DM.

## Conclusion

Conclusively, the study demonstrates a low prevalence of HBV and HCV among patients with DM as compared to the general population, with no significant association among factors. However, there is still a need for screening HBV and HCV in the general population, especially in the vulnerable population. This study potentiates the importance of early screening for HBV and HCV, as well as HBV vaccination, in patients with DM.
